# Adrenal enlargement and failure of suppression of circulating cortisol by dexamethasone in patients with malignancy

**DOI:** 10.1038/sj.bjc.6690603

**Published:** 1999-08

**Authors:** P J Jenkins, S A Sohaib, P J Trainer, T A Lister, G M Besser, R Reznek

**Affiliations:** Departments of Endocrinology, Radiology, ICRF Dept of Medical Oncology, St Bartholomew's Hospital, West Smithfield, London, EC1A 7BE, UK

## Abstract

The aim of this study was to further elucidate the activity of the hypothalamo–pituitary–adrenal (HPA) axis in patients with malignancy and to correlate this with the size of the adrenal glands. Fourteen patients with a variety of malignancies were studied prior to receiving cytotoxic chemotherapy. During routine staging computerized tomographic (CT) scans, the size of the body, medial and lateral limbs of the adrenal glands were measured and compared with those of a normal group of patients studied previously. Measurements of 09:00 h serum cortisol and plasma adrenocorticotropic hormone (ACTH) levels were made before and after the administration of dexamethasone (0.5 mg 6-hourly for 48 h) in addition to the peak cortisol response to i.v corticotropin releasing hormone (CRH). Overall, patients with malignancy had significantly larger adrenal glands than patients without malignancy; those with non-haematological malignancies had larger glands than patients with haematological malignancies. Following dexamethasone to suppress circulating cortisol levels, nine patients (64%) demonstrated abnormal resistance with cortisol levels > 50 nmol l^−1^: mean value 294 nmol l^−1^ (range 67–1147). Those patients who failed to suppress after dexamethasone had significantly larger adrenal glands than those that did suppress and tended to have non-haematological malignancies. ACTH levels were undetectable or low in three patients in whom it was measured and who did not suppress with dexamethasone. Following CRH, the cortisol levels were highest (823 and 853 nmol l^−1^) in two of these patients. Malignancy is associated with diffuse enlargement of the adrenal glands and resistance to dexamethasone-induced suppression of the HPA axis, which is not due to ectopic ACTH secretion. This disturbance of the normal control of the HPA axis is unexplained and its functional significance remains uncertain. © 1999 Cancer Research Campaign


*British Journal of Cancer* (1999) **80**(11), 1815–1819
© 1999 Cancer Research Campaign
Article no. bjoc.1999.0603

### Adrenal enlargement and failure of suppression of circulating cortisol by dexamethasone in patients with malignancy

**PJ Jenkins[1], SA Sohaib[2], PJ Trainer[1], TA Lister[3], GM Besser[1] and R Reznek[2]**

Departments of [1]Endocrinology, [2]Radiology and [3]ICRF Dept of Medical Oncology, St Bartholomew's Hospital, West Smithfield, London EC1A 7BE, UK

**Summary** The aim of this study was to further elucidate the activity of the hypothalamo–pituitary–adrenal (HPA) axis in patients with malignancy and to correlate this with the size of the adrenal glands. Fourteen patients with a variety of malignancies were studied prior to receiving cytotoxic chemotherapy. During routine staging computerized tomographic (CT) scans, the size of the body, medial and lateral limbs of the adrenal glands were measured and compared with those of a normal group of patients studied previously. Measurements of 09:00 h serum cortisol and plasma adrenocorticotropic hormone (ACTH) levels were made before and after the administration of dexamethasone (0.5 mg 6-hourly for 48 h) in addition to the peak cortisol response to i.v corticotropin releasing hormone (CRH). Overall, patients with malignancy had significantly larger adrenal glands than patients without malignancy; those with non-haematological malignancies had larger glands than patients with haematological malignancies. Following dexamethasone to suppress circulating cortisol levels, nine patients (64%) demonstrated abnormal resistance with cortisol levels > 50 nmol $l^{-1}$: mean value 294 nmol $l^{-1}$ (range 67–1147). Those patients who failed to suppress after dexamethasone had significantly larger adrenal glands than those that did suppress and tended to have non-haematological malignancies. ACTH levels were undetectable or low in three patients in whom it was measured and who did not suppress with dexamethasone. Following CRH, the cortisol levels were highest (823 and 853 nmol $l^{-1}$) in two of these patients. Malignancy is associated with diffuse enlargement of the adrenal glands and resistance to dexamethasone-induced suppression of the HPA axis, which is not due to ectopic ACTH secretion. This disturbance of the normal control of the HPA axis is unexplained and its functional significance remains uncertain.

It has previously been reported that patients with lymphoma and other malignant tumours have significantly larger adrenal glands than normal subjects (Vincent et al, 1994*b*). This enlargement is due to hyperplasia rather than metastatic deposits, and is unrelated to the stage of disease. However, its clinical significance and relation to adrenocortical function remain undetermined. Two early studies on adrenocortical function in patients with malignancy reported conflicting results. One demonstrated diminished urinary adrenocortical steroid excretion (Dobriner et al, 1950), whilst another reported increased basal secretion and impaired suppression following the administration of a single dose of dexamethasone (Bishop and Ross, 1971). However, in the latter study, the majority of patients had an oat cell bronchogenic carcinoma and thus it is probable that their adrenocortical overactivity was due to ectopic adrenocorticotropic hormone (ACTH) production. This was unlikely to be responsible for the report of adrenal enlargement in patients with malignancy by Vincent et al (1994*b*): not only did none of the patients have or develop clinical stigmata of the ectopic ACTH syndrome, but this condition is only rarely associated with haematological malignancies, which made up a large number of the studied patients. The aims of the present study were to elucidate the activity of the hypothalamo–pituitary–adrenal (HPA) axis in patients with malignancy and to correlate this with the size of adrenal glands, as measured by computerized tomography (CT), and to test whether any abnormalities were likely or not to be due to ectopic ACTH production.

*Received 10 August 1998*
*Revised 4 January 1999*
*Accepted 20 February 1999*

*Correspondence to:* PJ Jenkins

### METHODS

Fourteen patients with a variety of malignancies were studied at their initial presentation before the administration of any chemotherapy or radiotherapy (Table 1). Patients with renal or hepatic failure, sepsis, or chronic inflammatory conditions were excluded and all the patients were ambulant and well; none were incapacitated by their disease. The study was approved by the local Ethical Committee and all patients gave written informed consent. The patients did not have any clinical stigmata of Cushing's syndrome and had not previously received glucocorticoids. After resting in a supine position for 30 min, a fasting serum blood sample was taken at 09:00 h (2 + 0). Each patient then received oral dexamethasone (0.5 mg 6-hourly for 48 h), and a further serum 09:00 h cortisol measurement was obtained after the last dose (2 + 48). Normally, serum cortisol levels after 48 h of dexamethasone (2 mg $day^{-1}$) are < 50 nmol $l^{-1}$.

A corticotrophin releasing hormone (CRH) stimulation test was performed in six patients on the day prior to the administration of the first dose of dexamethasone. After fasting from midnight, an intravenous cannula was inserted and the patient rested in a supine position for 30 min. At 09:00 h, 100 μg of human CRH were administered and serum cortisol measurements taken 15 min before, and every 15 min afterwards for 2 h. In normal subjects, the maximum cortisol level after hCRH is 700 nmol $l^{-1}$ (Trainer et al, 1995).

**Table 1** The clinical details of the patients studied

| Patients | Sex | Age | Diagnosis | Stage |
|---|---|---|---|---|
| 1 | F | 39 | Nodular sclerosing HD | I A |
| 2 | M | 27 | Nodular sclerosing HD | I A |
| 3 | M | 72 | High grade B-cell NHL | II B |
| 4 | M | 45 | High grade T-cell NHL | II E |
| 5 | M | 73 | High grade B-cell NHL | II A |
| 6 | M | 26 | Nodular sclerosing HD | III A |
| 7 | F | 61 | Low grade B-cell NHL | IV A |
| 8 | M | 75 | Low grade B-cell NHL | IV A |
| 9 | M | 85 | Rectal carcinoma | |
| 10 | M | 85 | Metastatic adenocarcinoma (unknown primary) | |
| 11 | M | | Oesophageal carcinoma | |
| 12 | M | 64 | Carcinomatosis (unknown primary) | |
| 13 | M | 76 | Renal carcinoma | |
| 14 | F | 72 | Pancreatic carcinoma | |

HD, Hodgkin's disease; NHL, non-Hodgkin's lymphoma.

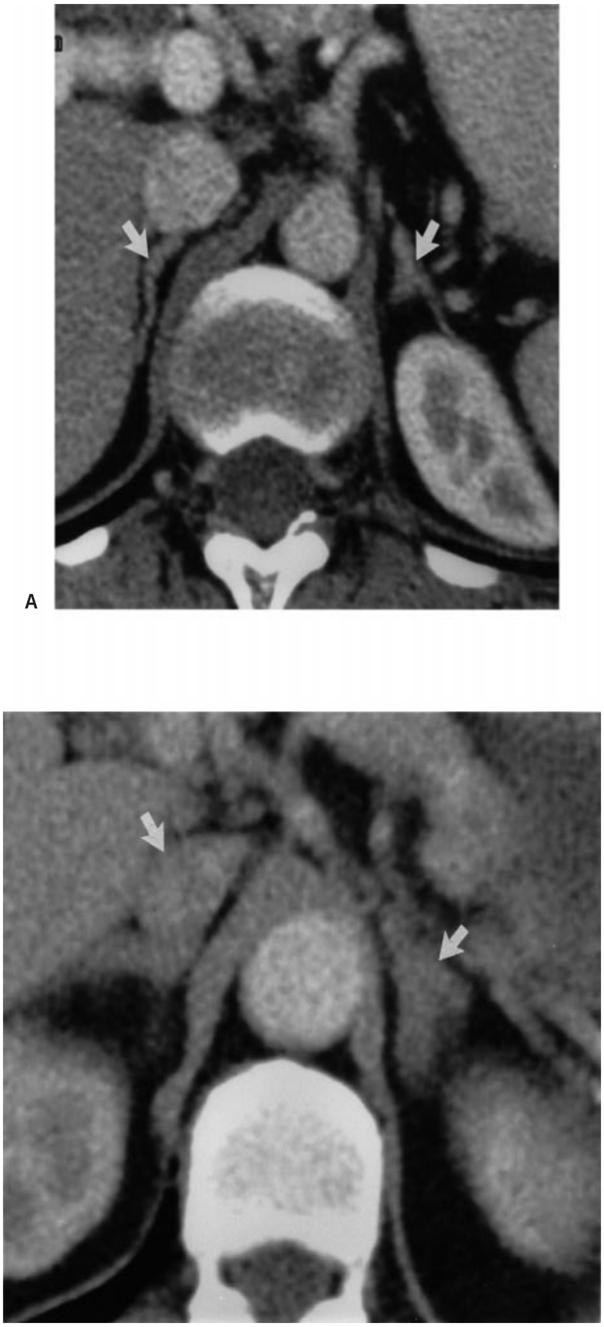

A

B

**Figure 1** CT images of normal adrenal glands (top; white arrows) and in a patient with malignancy (bottom; white arrows) showing diffuse enlargement

Serum cortisol was measured using an in-house radioimmunoassay (RIA) with a minimum detectable concentration set at 50 nmol $l^{-1}$ (inter-assay and intra-assay variation < 10%). A simultaneous plasma ACTH measurement was performed in six patients using an in-house RIA with a minimum detectable concentration of 10 pg $ml^{-1}$ (inter-assay and intra-assay variation < 10%).

CT scans were obtained as part of each patient's clinical staging using a GE 9800 Advantage system with standard abdominal window settings (window level 40, width 400). Contiguous 10-mm thick images were obtained and the best images showing each part of the adrenal gland were selected independently by two observers (SAS and RR) in a blinded manner. Using the standard GE 9800 measurement function, a cursor was deposited on the margins of the adrenal gland and measurements obtained. The measurements recorded were:

1. Maximum width of the gland: defined as the maximum width perpendicular to the long axis of the body of the gland, at the junction of the adrenal limbs and the body.
2. Width of the adrenal limbs: defined as the maximum width (thickness) of the medial and lateral limbs of the gland perpendicular to the long axis of the limb.

The measurements were compared to our previously established normal range for patients without malignancy or inflammatory conditions (Vincent et al, 1994*a*). The mean coefficient of variation for the inter-observer error of each measurement was 5.5–11.4%. In addition, images of the liver were visualized in each patient and the presence of hepatic metastasis excluded.

### Statistics

Student's *t*-test was used for comparisons of adrenal size between all the patients with malignant disease and normal controls; the Mann–Whitney test was used for comparison of adrenal size between patients with malignancy. A *P*-value of < 0.05 was taken as indicating statistical significance.

### RESULTS

The average measurements for the right and left adrenal glands and their comparison with normal values are shown in Table 2. Both adrenal glands were identified in all patients, but not all measurements could be made in every patient because of a paucity of retroperitoneal fat and/or contiguity with adjacent structures in some. The right lateral limb was not measured in three patients, the right medial in a further patient and the left lateral in an additional single patient. Patients with malignancy had highly significant enlargement of all measures of their adrenal glands compared to normal patients (Figure 1; Table 2).

*© 1999 Cancer Research Campaign*

**Table 2** The mean (± s.d.) measurements (mm) of the body, medial and lateral limbs of 14 patients with malignancy compared to normal controls

| | Right | | | Left | | |
|---|---|---|---|---|---|---|
| | Body | Medial limb | Lateral limb | Body | Medial limb | Lateral limb |
| Patients with malignancy | 7.6 (2.9) | 5.5 (1.7) | 4.8 (1.1) | 9.4 (2.7) | 6.3 (1.6) | 6.8 (2.1) |
| Normal control subjects | 6.1 (2) | 2.8 (0.8) | 2.8 (0.6) | 7.9 (2.1) | 3.3 (0.9) | 3.0 (1) |
| *P*-value | < 0.02 | < 0.0001 | < 0.0001 | < 0.02 | < 0.0001 | < 0.0001 |

**Table 3** The serum cortisol nmol $l^{-1}$ and plasma ACTH pgml$^{-1}$ values and size (mm) of the body, medial and lateral limbs of the adrenal glands in patients with a variety of malignancies, before and after a 48-h low-dose dexamethasone suppression test

| Patient | Cortisol 2 + 0 | Cortisol 2 + 48 | ACTH 2 + 0 | ACTH 2 + 48 | Right body | Right medial limb | Right lateral limb | Left body | Left medial limb | Left lateral limb |
|---|---|---|---|---|---|---|---|---|---|---|
| 1 | 417 | < 50 | 17 | < 10 | 5 | 4 | 5 | 7 | 4 | 5 |
| 2 | 390 | < 50 | 15 | < 10 | 7 | 4 | 5 | 6 | 5 | 5 |
| 3 | 814 | 110 | NR | NR | 8 | 5 | 4 | 11 | 8 | 10 |
| 4 | 928 | 98 | < 10 | < 10 | 4 | 6 | NV | 8 | 6 | 6 |
| 5 | 242 | < 50 | 14 | < 10 | 3 | NV | 3 | 5 | 4 | 4 |
| 6 | 322 | < 50 | NR | NR | 5 | 5 | 4 | 8 | 6 | 4 |
| 7 | 740 | < 50 | NR | NR | 9 | 4 | 3 | 10 | 6 | 6 |
| 8 | 525 | 67 | 29 | 11 | 5 | 4 | 5 | 9 | NV | 7 |
| 9 | 588 | 182 | NR | NR | 12 | 7 | NV | 8 | 7 | 7 |
| 10 | 545 | 222 | NR | NR | 8 | 9 | 6 | 14 | 8 | 9 |
| 11 | 983 | 1147 | NR | NR | 11 | 7 | 6 | 10 | 9 | 8 |
| 12 | 616 | 492 | NR | NR | 9 | 4.5 | 6 | 10 | 6 | 8 |
| 13 | 449 | 101 | 20 | 13 | 9 | 4 | NV | 12 | 5 | 5 |
| 14 | 598 | 232 | NR | NR | 12 | 8 | 6 | 14 | 8 | 11 |

NR, not recorded; NV, not visualized.

**Table 4** The mean (± s.e.m.) measurements (mm) of the body, medial and lateral limbs of the adrenal glands in patients with malignancy who failed to suppress serum cortisol levels to < 50 nmol $l^{-1}$ after a 48-h low-dose dexamethasone suppression test compared to those patients who did suppress

| | Right | | | Left | | |
|---|---|---|---|---|---|---|
| | Body | Medial limb | Lateral limb | Body | Medial limb | Lateral limb |
| Non-suppression | 8.7 (2.8) | 6.1 (1.8) | 5.5 (0.8) | 10.7 (2.3) | 7.1 (1.4) | 7.9 (1.9) |
| Suppression | 5.8 (2.2) | 4.2 (0.5) | 4.0 (1) | 7.2 (1.9) | 5.0 (1) | 4.8 (0.8) |
| *P*-value | NS | NS | 0.03 | 0.02 | 0.02 | 0.01 |

NS, not significant.

Patients with non-haematological malignancies had significantly larger glands compared to patients with haematological malignancies (right body $P < 0.005$; left body $P < 0.03$; right lateral limb $P < 0.01$; right medial limb $P < 0.05$) and had significantly higher cortisol levels after administration of dexamethasone ($P < 0.002$). There was no relationship between the adrenal size and stage of haematological disease.

For all patients the range of serum cortisol values before dexamethasone was 242–983 nmol $l^{-1}$ (median 545 nmol $l^{-1}$) and after its administration < 50–1147 nmol $l^{-1}$ (median 98 nmol $l^{-1}$)

© 1999 Cancer Research Campaign respectively (Table 3). Following dexamethasone, nine patients (64%) had cortisol levels above 50 nmol $l^{-1}$ reflecting resistance to suppression: mean value 294 nmol $l^{-1}$, range 67–1147. The 2+48 ACTH levels in the three of these in whom it was measured were < 10, 11 and 13 pg $ml^{-1}$; it was < 10 pg $ml^{-1}$ in three other patients in whom it was measured, each of whom had cortisol levels < 50 nmol $l^{-1}$ after dexamethasone. Those patients who failed to suppress circulating cortisol after dexamethasone had significantly larger adrenal glands compared to those who did suppress (Table 4).

The maximum cortisol level following CRH ranged from 481 to 853 nmol $l^{-1}$ (median 616) and the percentage increment from –3 to 46% (median 32%). The maximum cortisol levels after CRH were highest (823 and 853 nmol $l^{-1}$) in two patients who failed to suppress with dexamethasone.

### DISCUSSION

This study has demonstrated that patients with a variety of malignancies have significantly enlarged adrenal glands as compared to normal patients. This is in accordance with our previous findings in a different group of patients, but who had a similar variety of malignant disease (Vincent et al, 1994*b*). However, in that study, no attempt was made to assess the functional activity of the adrenal glands and to correlate it with the adrenal size. These results have shown that not only is malignancy associated with elevated levels of cortisol that are often resistant to suppression with dexamethasone, but that such resistance is associated with larger adrenal glands. That this enlargement is not due to metastatic deposits is indicated by the diffuse nature of the enlargement without any focal nodules, the lack of any difference in size of the adrenal glands between patients with early or advanced disease and the presence of adrenal enlargement in patients with a wide variety of tumours, including lymphoma, which involves the adrenals only rarely. In addition, metastatic enlargement would not be expected to result in hyperfunctioning of the gland. Although we cannot definitely exclude microscopic metastases as the cause for the enlargement, such an occurrence would not be expected to result in diffuse enlargement.

These findings of increased adrenocortical activity concur with those of an earlier study by Bishop and Ross (1971). They studied patients with surgically inoperable and metastatic cancer, particularly those with oat cell bronchial carcinoma. Although there was no significant difference in the 09:00 h plasma 11-hydroxycorticoid (mainly cortisol) concentrations between normal patients and those with either oat cell carcinoma or other tumours, both groups of patients with malignancy had significantly elevated midnight steroid values and exhibited less suppression following the administration of a single 2 mg dose of dexamethasone. However, the patients in this study differed from those reported above, not only in the more advanced nature of their disease, but also in that it is probable that most if not all of their adrenocortical hyperfunction was due to ectopic ACTH production, a common feature of bronchogenic oat cell carcinomas. Such ectopic secretion by the tumours is unlikely to be responsible for our findings. The maximum level of ACTH, in the six patients in whom it was measured (three of whom failed to suppress after dexamethasone), before and after dexamethasone was 29 pg $ml^{-1}$ and 13 pg $ml^{-1}$ respectively. These levels are lower than those recognized to be associated with ectopic production of ACTH, characteristically greater than 200 pg $ml^{-1}$ (Besser and Landon, 1968; Ratcliffe et al, 1972; Pullan et al, 1980; Howlett et al, 1986) and suggest an alternative pathogenesis for the hyperplasia and resistance to dexamethasone. An ectopic source of ACTH is also discounted by the rarity of this syndrome being recorded with haematological malignancies which accounted for the majority of our patients. The data from the CRH tests also suggests an ectopic source of ACTH is less likely. Apart from one patient who suppressed with dexamethasone and who had a flat response to CRH, the remaining patients had a percentage rise above basal cortisol values of between 26% and 46%. This is greater than the flat response that is characteristic of patients with Cushing's syndrome due to ectopic ACTH (Grossman et al, 1988). The responses to CRH exhibited by our patients also exclude autonomous adrenal functioning, as such patients exhibit a flat response to this peptide (Grossman et al, 1988). Thus, the combination of low levels of ACTH and the normal or exaggerated cortisol responses to CRH suggest the action of (i) either an alternative non-ACTH-mediated stimulant acting predominantly independently on the adrenals or synergistically on the adrenal response to ACTH, or (ii) acquired resistance to dexamethasone at the pituitary and/or adrenal level. One pathogenic mechanism might relate to perturbations in the homeostatic interplay between the HPA axis and the immune system, in the form of cytokines (Jenkins and Grossman, 1997). It has been clearly established that interleukins (ILs) 1α, IL-1β, IL-6 and IL-2 are all able to exert a direct stimulatory effect on adrenocortical cortisol secretion (Roh et al, 1987; Whitcomb et al, 1988; Salas et al, 1990; Tominaga et al, 1991; O'Connell et al, 1994), with the stimulatory effects of IL-1α and IL-1β being suggested to be mediated via prostaglandins ($PGD_2$, $PGF_2$ and $PGE_2$) (Winter et al, 1990; Tominaga et al, 1991). Furthermore, IL-6 has been shown to act synergistically with physiological concentrations of ACTH in stimulating cortisol secretion (Salas et al, 1990). Conversely, there is increasing evidence that inflammatory conditions which favour the release of cytokines are associated with localized resistance to glucocorticoids and antagonism to their suppressive effects, perhaps by increasing the expression of the non-ligand-binding β-isoform of the glucocorticoid receptor (de Castro et al, 1996; Lamberts et al, 1996). A number of these cytokines, including γ-interferon and tumour necrosis factor-α, may be secreted by tumours (Merz et al, 1991) and indeed the secretion of IL-6 has recently been shown to correlate with disease activity and to be associated with adverse prognostic features (Seymour et al, 1995). Furthermore, the experimental in vivo transplantation of lymphoma cells has been shown to markedly elevate glucocorticoid levels, an effect that can be mirrored by the transference of ascitic fluid from the lymphoma-bearing animal (Normann et al, 1988*a*, 1988*b*).

Whatever the precise mechanism underlying both the adrenal enlargement and disturbed pituitary–adrenal regulation, the question arises as to its functional significance. The development of a malignancy might result in increased cytokine levels either via activation of the immune system due to the associated antigenic changes or from direct secretion by the tumour. Resultant interference with the HPA axis, perhaps via cytokine-mediated localized resistance to glucocorticoids might aid tumour growth by minimizing their immunosuppressive effects. Such a hypothesis is supported by the clinical benefits of high-dose glucocorticoid therapy as an effective constituent of the treatment of lymphoma; although, conversely, patients with ectopic ACTH resulting from oat cell carcinomas have a worse prognosis compared to non-ACTH secretors (Hymes and Doe, 1962; Dimopoulos et al, 1992;

© 1999 Cancer Research Campaign

Shepherd et al, 1992). Clarification of the clinical significance of these findings awaits prospective longitudinal studies comparing adrenal size and function with clinical outcome. Until these issues are resolved, a radiological report of diffuse bilateral adrenal gland enlargement in a patient with a known carcinoma and in the absence of any clinical stigmata of Cushing's syndrome, probably does not required detailed endocrinological investigation.

### ACKNOWLEDGEMENTS

Dr Paul Jenkins was supported by the Special Trustees of St Bartholomew's Hospital during the duration of this study. Prof. TA Lister is supported by the Imperial Cancer Research Fund. The authors are very grateful to the research nurses in the Department of Endocrinology, the radiographers in the Department of Radiology, the chartists in the Department of Medical Oncology and the other clinicians who permitted the study of their patients. The CRH was a generous gift from Ferring, Kiel, Germany.

### REFERENCES

Besser GM and Landon J (1968) Plasma levels of immunoreactive corticotrophin in patients with Cushing's syndrome. *Br Med J* **4**: 552–554

Bishop MC and Ross EJ (1971) Adrenocortical activity in disseminated malignant disease in relation to prognosis. *Br J Cancer* **25**: 719–725

de Castro M, Elliot S, Kino T, Bamberger C, Karl M, Webster E and Chrousos GP (1996) The non-ligand binding beta-isoform of the human glucocorticoid receptor (hGR beta): tissue levels, mechanism of action, and potential physiologic role. *Mol Med* **2**: 597–607

Dimopoulos MA, Fernandez JF, Samaan NA, Holoye PY and Vassilopoulou-Sellin R (1992) Paraneoplastic Cushing's syndrome as an adverse prognostic factor in patients who die early with small cell lung cancer. *Cancer* **69**: 66–71

Dobriner K, Lieberman S, Wilson H, Ekman B, Pearson O and Eliel L (1950) In: Mote JR (ed) *Proceedings of the First Clinical ACTH Conference*. pp. 158–167. Blakiston: Philadelphia

Grossman AB, Howlett TA, Perry L, Coy DH, Savage MO, Lavender P, Rees LH and Besser GM (1988) CRF in the differential diagnosis of Cushing's syndrome: a comparison with the dexamethasone suppression test. *Clin Endocrinol* **29**: 167–178

Howlett TA, Drury PL, Perry L, Doniach I, Rees LH and Besser GM (1986) Diagnosis and management of ACTH-dependent Cushing's syndrome: comparison of the features in ectopic and pituitary ACTH production. *Clin Endocrinol* **24**: 699–713

Hymes AC and Doe RP (1962) Adrenal function in cancer of the lung, with and without Cushing's syndrome. *Am J Med* **33**: 398–407

Jenkins PJ and Grossman A (1997) *Neuroimmunoendocrinology* In: Sheaves R, Jenkins PJ and Wass J (eds) *Clinical Endocrine Oncology*. Blackwell Science: Oxford

Lamberts SW, Huizenga AT, de Lange P, de Jong FH and Koper JW (1996) Clinical aspects of glucocorticoid sensitivity. *Steroids* **61**: 157–160

Merz H, Fliedner A, Orscheschek K, Binder T, Sebald W, Muller-Hermelink HK and Feller AC (1991) Cytokine expression in T-cell lymphomas and Hodgkin's disease. Its possible implication in autocrine or paracrine production as a potential basis for neoplastic growth. *Am J Pathol* **139**: 1173–1180

Normann S, Besedovsky H, Schardt M and del Ray A (1988a) Interactions between endogenous glucocorticoids and inflammatory responses in normal and tumor-bearing mice: role of T cells. *J Leukoc Biol* **44**: 551–558

Normann S, Besedovsky H, Schardt M and del Rey A (1988b) Hormonal changes following tumor transplantation: factors increasing corticosterone and the relationship of corticosterone to tumor-induced anti-inflammation. *Int J Cancer* **41**: 850–854

O'Connell NA, Kumar A, Chatzipanteli K, Mohan A, Agarwal RK, Head C, Bornstein SR, Abou-Samra AB and Gwosdow AR (1994) Interleukin-1 regulates corticosterone secretion from the rat adrenal gland through a catecholamine-dependent and prostaglandin E2-independent mechanism. *Endocrinology* **135**: 460–467

Pullan PT, Clement-Jones V, Corder R, Lowry PJ, Besser GM and Rees LH (1980) ACTH LPH and related peptides in the ectopic ACTH syndrome. *Clin Endocrinol* **13**: 437–445

Ratcliffe JG, Knight RA, Besser GM, Landon J and Stansfeld AG (1972) Tumor and plasma ACTH concentrations in patients with and without the ectopic ACTH syndrome. *Clin Endocrinol* **1**: 27–44

Roh MS, Drazenovich KA, Barbose JJ, Dinarello CA and Cobb CF (1987) Direct stimulation of the adrenal cortex by interleukin-1. *Surgery* **102**: 140–146

Salas MA, Evans SW, Levell MJ and Whicher JT (1990) Interleukin-6 and ACTH act synergistically to stimulate the release of corticosterone from adrenal gland cells. *Clin Exp Immunol* **79**: 470–473

Seymour JF, Talpaz M, Cabanillas F, Wetzler M and Kurzrock R (1995) Serum interleukin-6 levels correlate with prognosis in diffuse large-cell lymphoma. *J Clin Oncol* **13**: 575–582

Shepherd FA, Laskey J, Evans WK, Goss PE, Johansen E and Khamsi F (1992) Cushing's syndrome associated with ectopic corticotropin production and small-cell lung cancer. *J Clin Oncol* **10**: 21–27

Tominaga T, Fukata J, Naito Y, Usui T, Murakami N, Fukushima M, Nakai Y, Hirai Y and Imura H (1991) Prostaglandin-dependent in vitro stimulation of adrenocortical steroidogenesis by interleukins. *Endocrinology* **128**: 526–531

Trainer PJ, Faria M, Newell-Price J, Browne P, Kopelman P, Coy DH, Besser GM and Grossman AB (1995) A comparison of the effects of human and ovine corticotropin-releasing hormone on the pituitary–adrenal axis. *J Clin Endocrinol Metab* **80**: 412–417

Vincent JM, Morrison ID, Armstrong P and Reznek RH (1994*a*) The size of normal adrenal glands on computed tomography. *Clin Radiol* **49**: 453–455

Vincent JM, Morrison ID, Armstrong P and Reznek RH (1994*b*) Computed tomography of diffuse, non-metastatic enlargement of the adrenal glands in patients with malignant disease. *Clin Radiol* **49**: 456–460

Whitcomb RW, Linehan WM, Wahl LM and Knazek RA (1988) Monocytes stimulate cortisol production by cultured human adrenocortical cells. *J Clin Endocrinol Metab* **66**: 33–38

Winter JS, Gow KW, Perry YS and Greenberg AH (1990) A stimulatory effect of interleukin-1 on adrenocortical cortisol secretion mediated by prostaglandins. *Endocrinology* **127**: 1904–1909

© 1999 Cancer Research Campaign
